# Radiological and chemical hazards of persistent organic pollutants in the textile sector

**DOI:** 10.1038/s41598-025-03581-9

**Published:** 2025-06-20

**Authors:** Sameh H. Fouda, E. S. Abd El-Halim, H. A. Abdel Ghany

**Affiliations:** Department of Physics, Faculty of Women for Arts, Science and Education, Ain-Shams University, Cairo, Egypt

**Keywords:** Gamma spectrometry, Radionuclides, Radiation hazards, Disperse, direct and reactive dyes, Dye wastewater, Worker safety, Textile industry, Environmental sciences, Physics

## Abstract

The textile industry exposes people to various harmful and allergenic compounds, with dye wastewater being a significant source of persistent organic pollutants (chemical substances accumulate in living organisms and pose risks to human health and ecosystems) in the environment. This study aimed to measure the activity concentrations of radionuclides, specifically ^238^U, ^226^Ra, ^232^Th, and ^40^K, in different types of textile dyes (disperse, direct, and reactive) and dye wastewater from the cities of Abour and Badr, using gamma spectrometry with a Hyper Pure Germanium detector. Additionally, heavy metal concentrations (Zn, Cd, Fe, Pb, Co, and K) were analyzed through Atomic Absorption Spectroscopy. The results indicated that the average specific activities of ^238^U, ^226^Ra, ^232^Th, and ^40^K were higher in disperse dyes compared to direct and reactive dyes. Potential radiation hazards were evaluated, revealing detectable levels of radioactivity in some textile dyes. This underscores the need for safety protocols and preventive measures for workers in the textile industry and those handling these dyes.

## Introduction

Textile dyes play a critical role in the fashion and textile industries. The use of dyes dates back thousands of years, with ancient civilizations like the Egyptians, Indians, and Chinese pioneering the art of dyeing textiles with natural sources such as plants, minerals, and insects. Over time, this practice evolved into a complex and scientifically advanced field that today relies heavily on synthetic dyes to meet the demands of mass production, environmental standards, and fast-changing consumer preferences^1^.

**Fig. 1:**
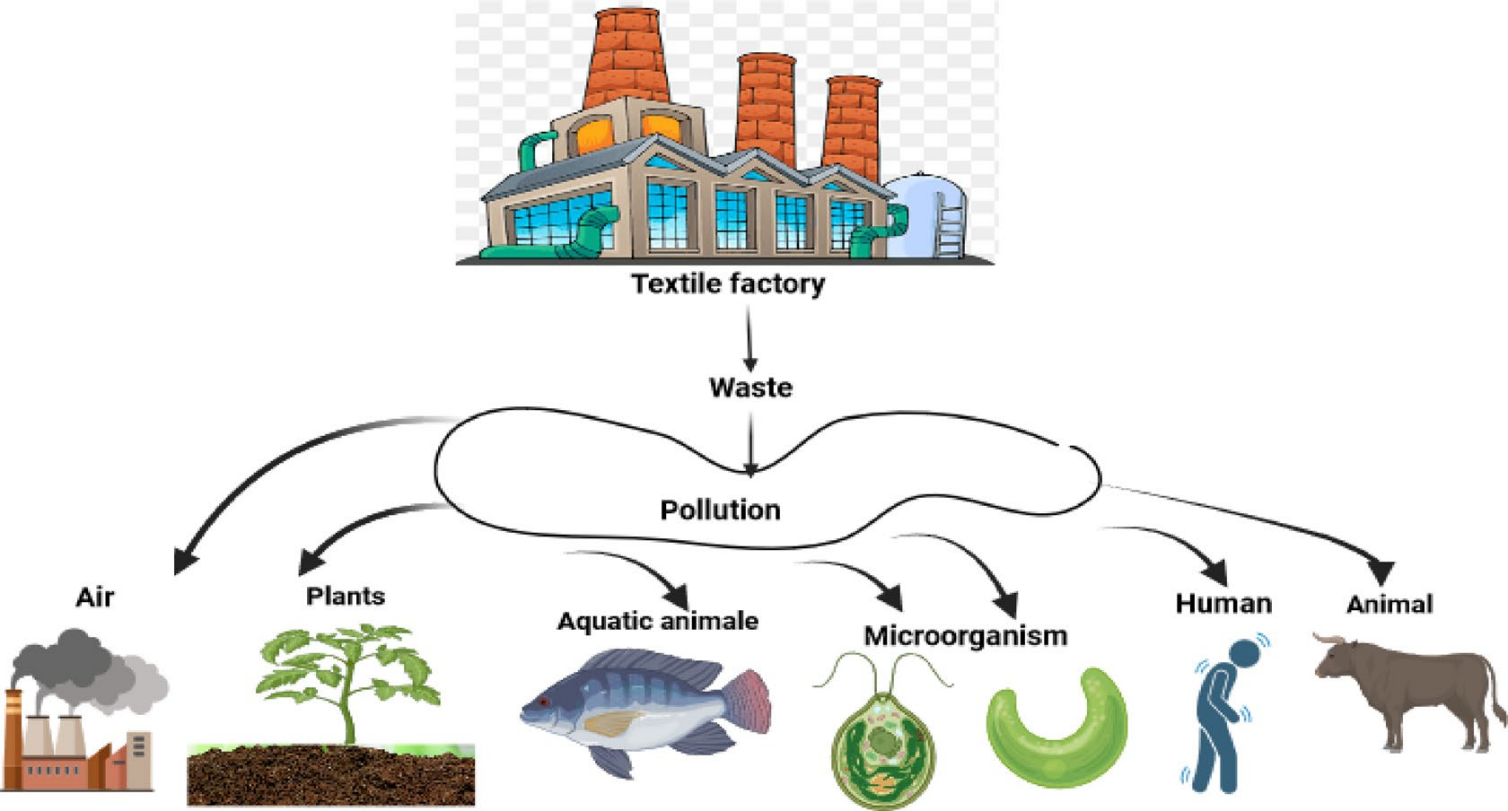
Forms of pollution from textile waste.

The textile industry is a significant contributor to environmental degradation, with a range of pollutants such as particulate matter, dust, sulfur and nitrogen oxides, and volatile organic compounds being released during textile dyeing processes^2^. The use of synthetic dyes poses serious environmental and health concerns. Textile dyes are known to contaminate large volumes of water, as they do not fully bind to fabrics and are often discharged as effluent into water bodies without proper treatment. This can harm aquatic life and disrupt ecosystems^3^, potentially introducing pollutants into the food chain (Fig. 1). Additionally, exposure to textile dyes—whether through ingestion, inhalation of dust, or skin contact—can cause acute toxicity, skin irritation, and eye problems^4^.

Various types of dyes are used in the textile industry, including reactive, disperse, and direct dyes, each with different applications and chemical properties. Reactive dyes, used on natural and synthetic fibers, form covalent bonds with fibers, while disperse dyes are used on synthetic materials and require high temperature and pressure to disperse and bond. Direct dyes, soluble in water, are commonly used on cellulose fibers like cotton and rayon, often requiring additional chemicals for better absorption. Furthermore, many textile processes, including dyeing and printing, make extensive use of heavy metals, which pose significant health risks when absorbed through the skin at elevated concentrations^5^.

Beyond chemical pollution, textile dyes and dyeing processes can introduce radioactive contaminants into the environment. Some raw materials used in dye production contain naturally occurring radionuclides such as uranium-238, radium-226, thorium-232, and potassium-40. These radionuclides can accumulate in textile effluents, posing potential risks to human health and environmental safety. Exposure to radiation from such contaminants can lead to long-term health effects, including carcinogenic risks and genetic mutations. Additionally, heavy metals such as lead, cadmium, chromium, and arsenic, which are commonly used as mordants or in pigment formulations, further exacerbate toxicity concerns. These elements persist in wastewater and soil, potentially contaminating agricultural produce and drinking water sources. Addressing these issues requires rigorous monitoring and regulatory measures to minimize the harmful effects associated with textile dyeing processes^6^. This study seeks to address the environmental and health concerns associated with these dyes by employing gamma spectrometry using a Hyper Pure Germanium detector, alongside atomic absorption spectroscopy. This approach aims to quantify levels of radionuclides such as uranium-238, radium-226, thorium-232, and potassium-40 as well as identify toxic elements present in textile dyes and dye wastewater^7^. By assessing these harmful substances, the study contributes to better understanding and mitigating the environmental and health impacts of the textile dyeing process.

## Experimental

### Ethics statement

The textile dyes collection and use were in accordance with all the relevant guidelines^8^.

### Sample preparation

#### Textile dyes

Eighteen textile dye samples—six disperses, eight reactive, and four direct, each in different colors were analyzed. These dyes, commonly used in Egypt’s textile industry, were sourced from local suppliers who imported them from Korea and China. To prepare the samples for gamma-ray analysis, they were dried in an oven at 110 °C for 3 h. (to remove moisture, ensuring that the dye mass is accurate). The dried samples were then placed in 200 ml cylindrical plastic containers with an inner diameter matching the detector’s face-to-face geometry. These containers were used to weigh and hermetically seal the samples. The packed samples were stored for four weeks to allow secular equilibrium to be established between ^226^Ra and ^232^Th and their decay products, minimizing radon escape^9^.

#### Dyes wastewater

Eight wastewater samples were collected from various dye factories: four from Obour City (labeled W1, W2, W3, and W4) and four from Badr City (labeled W5, W6, W7, and W8). The samples were gathered in identical 250 cm³ polyethylene beakers, which were also used for subsequent measurements. Each beaker was filled completely, with airtight caps ensuring the absence of air. The samples were then stored for over a month to allow the radioactive decay products to reach equilibrium with their parent isotopes, a crucial step to ensure radon gas remained confined within the sample. After the storage period, the activity concentration of radionuclides in the samples was measured using a high-purity Germanium detector (model GEM-15190).

#### Activity measurements

**Fig. 2:**
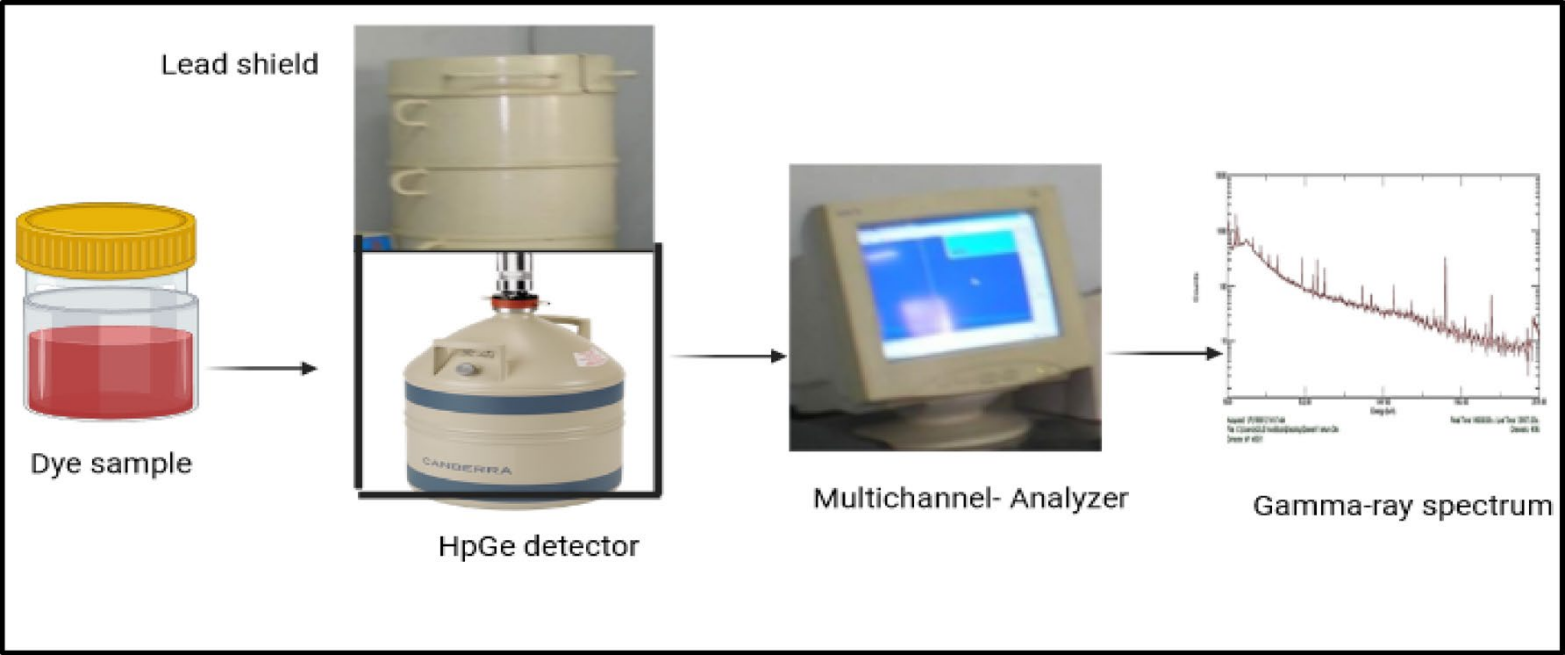
Gamma-ray system.

A high-resolution gamma detection system (in the physics laboratory at the Faculty of Women, Ain Shams University) was employed for radiometric analysis, utilizing an ORTEC high-purity germanium (HPGe) coaxial detector (model GEM-15190, serial No. 27-p-1876 A) (Fig. 2). The detector operates at a recommended bias voltage of -3 kV, with a crystal measuring 47.1 mm in length and 49.3 mm in diameter. The HPGe detector offers a full width at half maximum (FWHM) of 0.9 keV at the 122 keV gamma transition of ⁵⁷Co and 1.9 keV at the 1332.5 keV transition of ⁶⁰Co. Gamma-ray spectra were processed and analyzed using MAESTRO-32 software. Energy calibration was performed using the KeV/Ch mode to detect any undetected gamma-ray energies. Sources ⁶⁰Co (1173.2 and 1332.5 keV) and ¹³⁷Cs (661.9 keV) were used for calibration^10^. Efficiency calibration for the HPGe detector was conducted using a ²²⁶Ra point source (The most intensive gamma rays of ^226^Ra in equilibrium with its daughters have been used (Saif 2009). The relative intensities of the photo-peaks corresponding to these gamma ray lines have been measured by the detector and calculated. The relative efficiency curve of the detector was made of 11 different energy values covering the energy range from 186.2 keV to 1764.4 keV), and the relative efficiency curve for 250 ml beakers was normalized with a solution of chemically pure potassium chloride in distilled water (The radionuclide potassium-40, in the naturally occurring potassium, is perhaps the most widely used “standardized” low level source for beta-particle and gamma ray emitters. Naturally potassium, containing 0.0118% of potassium − 40, has a specific activity of about 850 pCi/g (31.45 Bq/g). The absolute efficiency curve was obtained using the same sample size^11^. The activity concentrations of ²³⁸U were calculated by measuring the 295.1 keV (19.2%) and 352 keV (37.2%) gamma rays from ²¹⁴Pb, as well as the 609.3 keV (46.1%) and 1120.3 keV (15.1%) gamma rays from ²¹⁴Bi. The ²³²Th activity was determined from gamma peaks at 238.6 keV (43.6%) from ²¹²Pb, 911.2 keV (29.0%) and 969.0 keV (23.2%) from ²²⁸Ac, and 583.0 keV (31.0%) from ²⁰⁸Tl. The concentration of ⁴⁰K was measured using its 1460 keV (10.7%) gamma line, while the concentration of ²²⁶Ra was determined by measuring its 186 keV (3.3%) gamma peak^12^. Each sample was analyzed over a detection period of 140,000 s, or roughly two days.

**Fig. 3:**
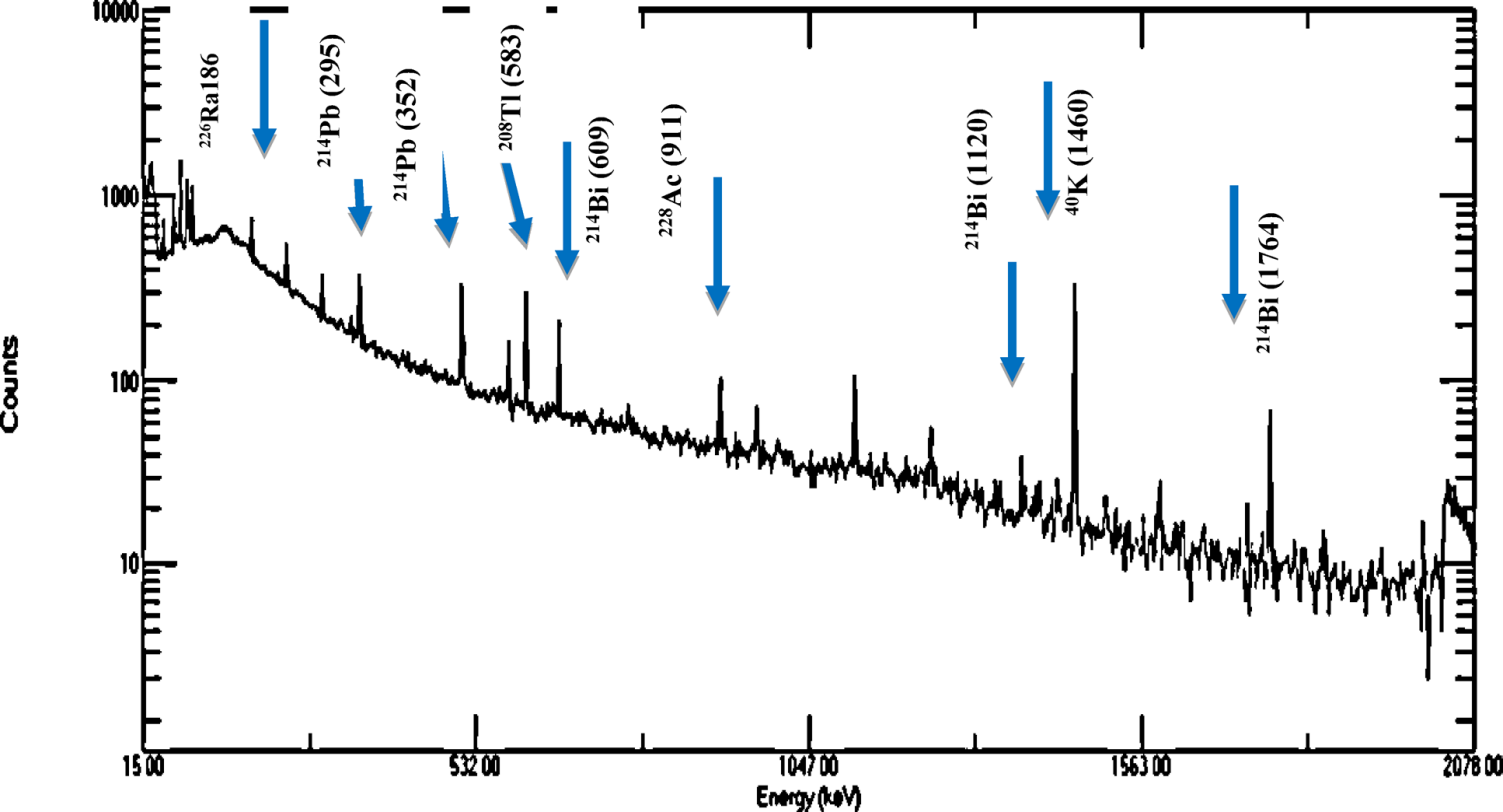
Gamma ray spectrum of the investigated black disperse dye sample.

### Atomic absorption measurements

In this study, eleven samples (three from disperse dye category, three from reactive dye category, one from direct dye category, two from Obour factory waste water category, two from Badr factory waste water category) of textile dyes and waste water samples are studied by atomic absorption spectroscopy to measure concentration of heavy metals including Cobalt (Co), Zinc (Zn), Cadmium (Cd), Iron (Fe), potassium (K), and Lead (Pb). Quality control and assurance in AAS involve strict adherence to standardized methods, proper calibration techniques, and regular validation using quality control samples. The minimum detection limits (MDL) vary by element and technique where the MDL for Cobalt (Co), Zinc (Zn), Cadmium (Cd), Iron (Fe), potassium (K), and Lead (Pb) are 0.05, 0.0033, 0.0028, 0.0043, 0.5 and 0.013 mg/kg respectively^13^. The material is exposed to a light beam in atomic absorption spectrometry. The amount of light that is absorbed depends on the element’s concentration. By comparing the intensity of the initial beam with the beam after passing the sample, one can estimate the element’s concentration. Since each element absorbs light with a certain wavelength, the AAS instruments have distinct light sources for each element. AAS is frequently used to identify only one ingredient per analysis^14,15^.

### Statistical analysis

Every measurement was taken in triplicate, and the average was then calculated. Following standard statistical procedures, the results were statistically examined using the SPSS 10.0 software package (SPSS, Chicago, IL, USA). The data was shown using means and standard deviations.

## Results

### Analysis of gamma spectrometry

**Fig. 4:**
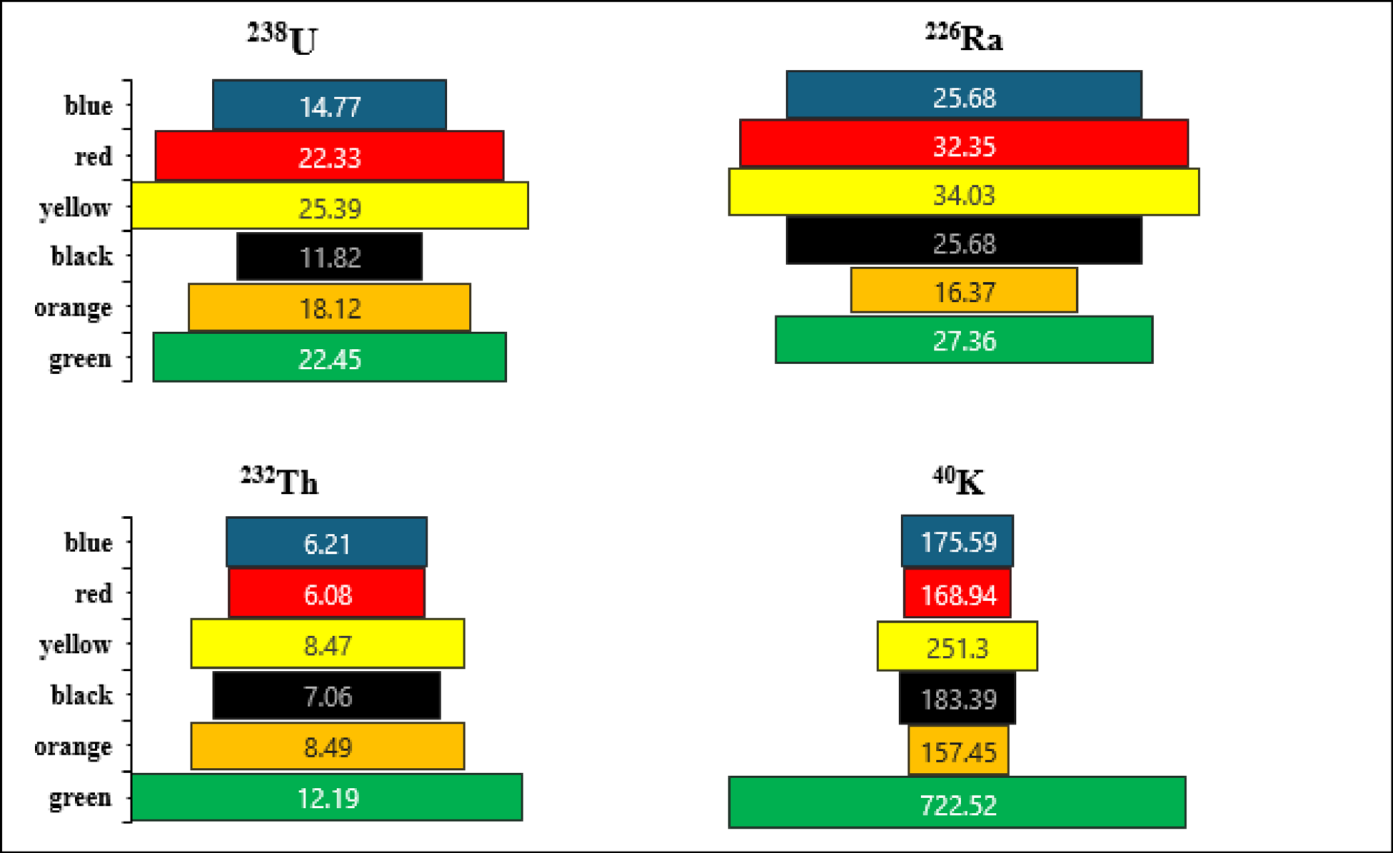
Specific activity (Bq/kg) of ^238^U, ^226^Ra, ^232^Th and ^40^ K in disperse dye samples.

**Fig. 5:**
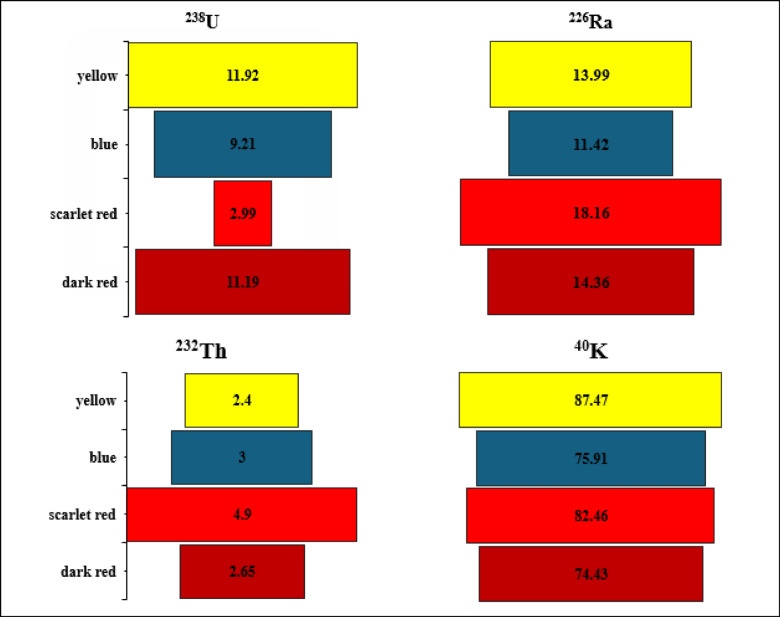
Specific activity of ^238^U, ^226^Ra, ^232^Th and ^40^ K in direct dye samples.

**Fig. 6:**
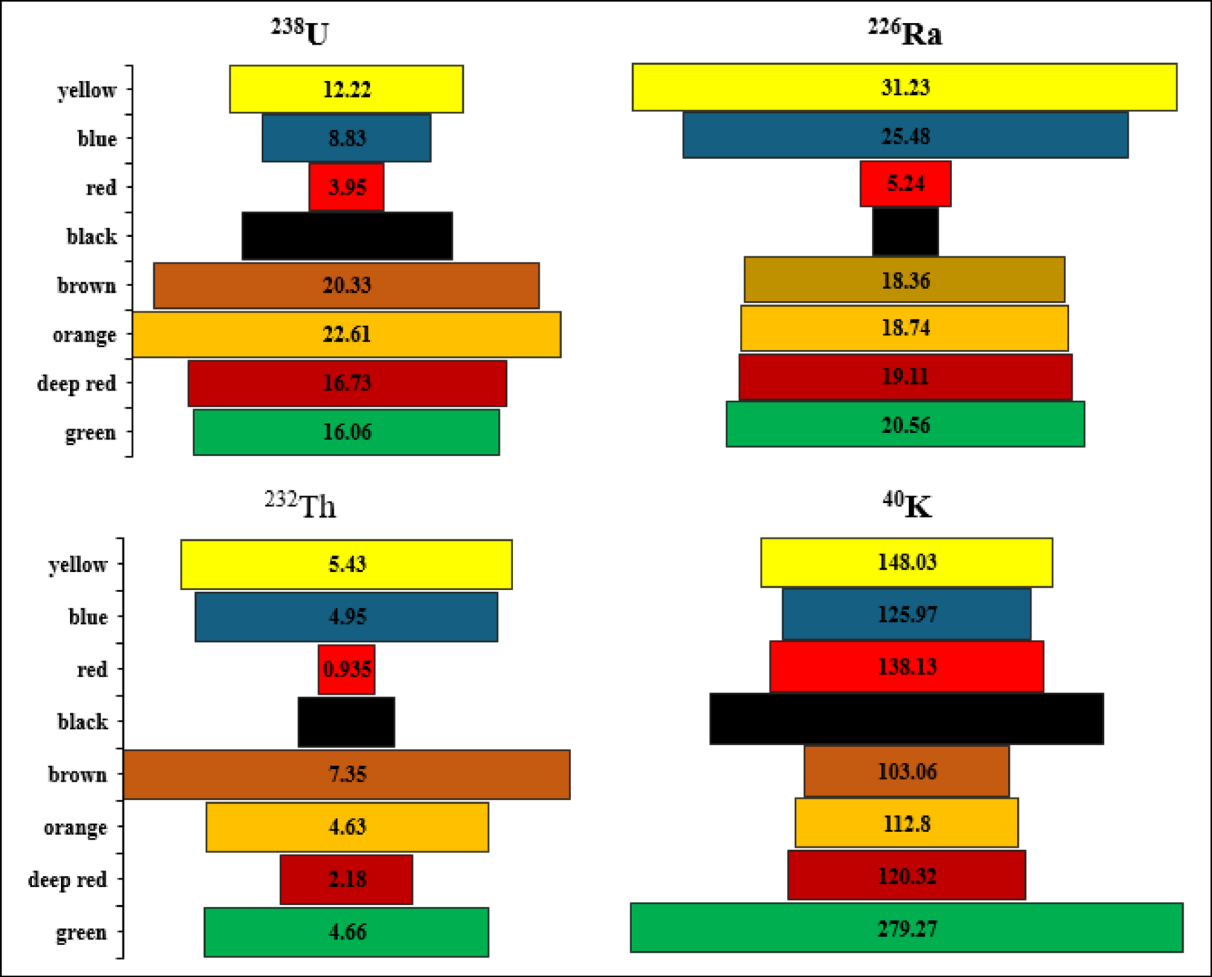
Specific activity of ^238^U, ^226^Ra, ^232^Th and ^40^ K in reactive dye samples.

**Fig. 7:**
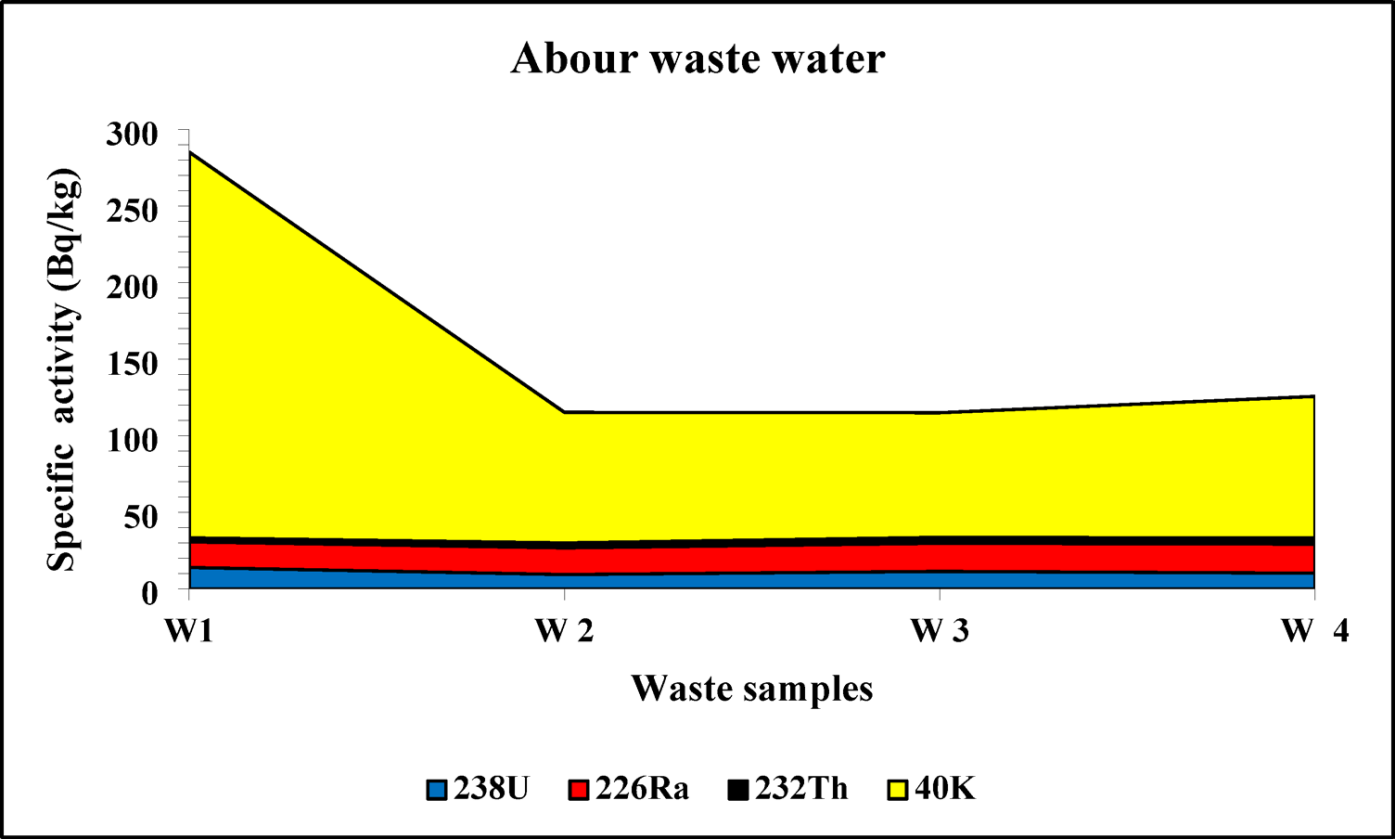
Specific activity of ^238^U, ^226^Ra, ^232^Th and ^40^ K in Abour wastewater samples.

**Fig. 8:**
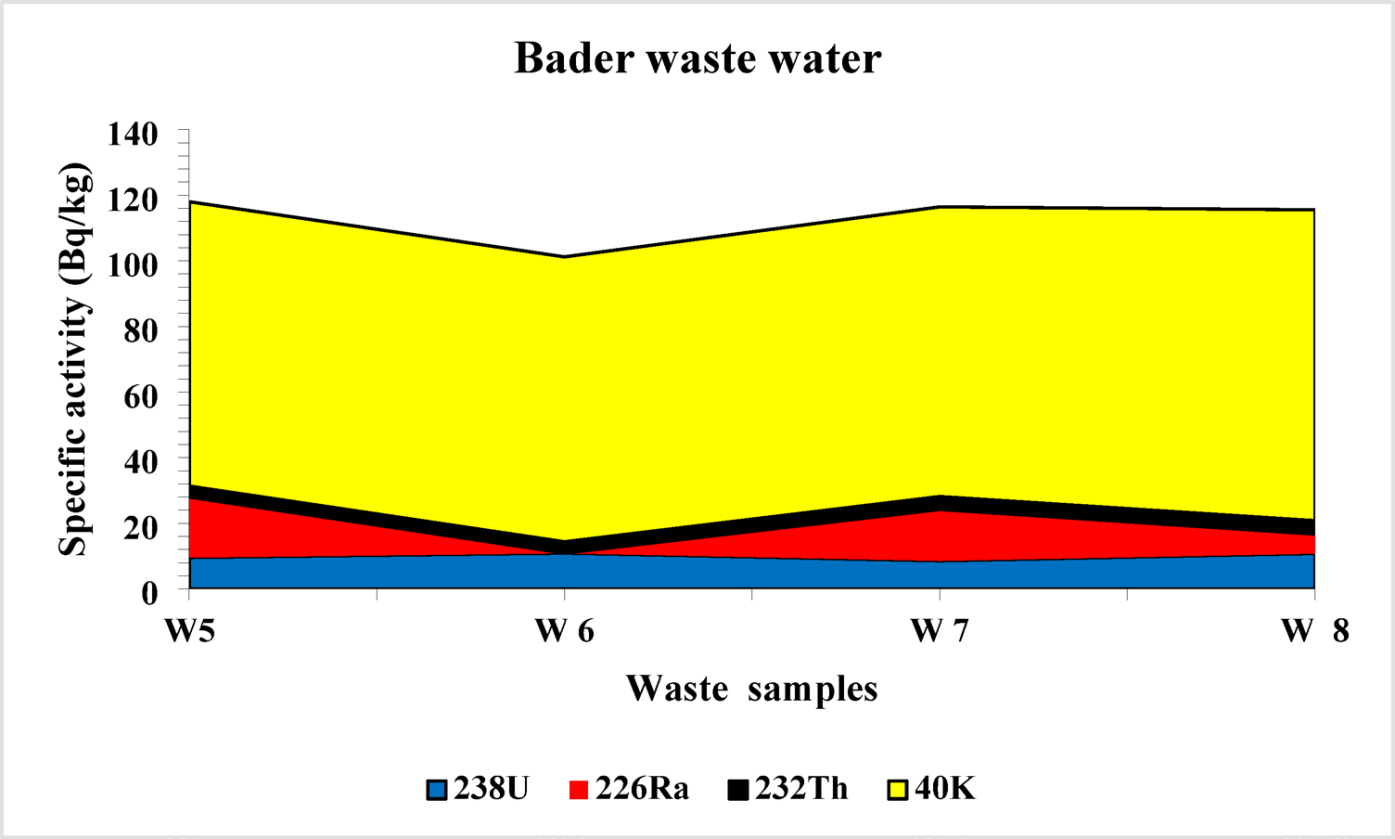
Specific activity of ^238^U, ^226^Ra, ^232^Th and ^40^ K in Bader wastewater samples.

The spectrum of the black disperse dye sample is shown on Fig. 3. The highest average concentrations of ^238^U, ^226^Ra, ^232^Th, and ^40^K in disperse dyes were 19.14 ± 5.17, 27.15 ± 6.23, 8.03 ± 2.26, and 276 ± 37 Bq/kg, respectively (Fig. 4). In contrast, the lowest average values were found in direct dyes, with ^238^U, ^226^Ra, ^232^Th, and ^40^K recording 8.82 ± 4.05, 14.48 ± 2.77, 3.23 ± 1.13, and 80.06 Bq/kg, respectively (Fig. 5). In reactive dyes, the ^238^U values ranged from 3.95 to 22.61 Bq/kg, while the ^226^Ra values ranged from 3.8 to 31.23 Bq/kg. The highest value of ^226^Ra (31.23 ± 7.16 Bq/kg) was observed in yellow dye, and the lowest value (3.8 ± 0.70 Bq/kg) in black dye. The lowest value of ^232^Th (0.935 ± 0.2 Bq/kg) was recorded in red dye, while the highest value (7.35 ± 2.0 Bq/kg) was found in brown dye. The mean specific activity of ^40^K was 153 ± 20 Bq/kg, with its highest value (279 ± 37 Bq/kg) in red dye and its lowest (87 ± 11 Bq/kg) in green dye (Fig. 6). This study marks the first time that natural radioactivity has been determined in dye wastewater. In Abour and Badr cities, where dye wastewater factories are located, the highest average values for ^238^U, ^226^Ra, ^232^Th, and ^40^K were recorded in Abour at 11.28 ± 1.2, 17.87 ± 3.3, 3.41 ± 0.9, and 127 ± 3.5 Bq/kg, respectively (Fig. 7). Meanwhile, in Badr, the respective values were 9.96 ± 1.9, 13.34 ± 1.2, 3.70 ± 0.9, and 89 ± 5.9 Bq/kg (Fig. 8).

### Radiological hazards

Various hazard indices were calculated for the samples investigated, including radium equivalent activity (Raeq), internal and external hazard indices (Hin, Hex), and outdoor and indoor excess lifetime cancer risk (ELCRout, ELCRin) as shown in Table 1. The results indicated that the lowest radium equivalent activity (Raeq) was 6.7 ± 0.13 Bqkg⁻¹ in direct dye (yellow sample), while the highest value of 100 ± 2.2 Bqkg⁻¹ was recorded in disperse dyes (green sample) (Table 2). All samples were below the maximum permissible limit of 370 Bqkg⁻¹^25^. The gamma index (Iγ) for the samples, presented in Table 2, ranged from 0.10 to 0.78 in disperse dyes, 0.08 to 0.36 in reactive dyes, and 0.06 to 0.22 in direct dyes all within acceptable limits. The external and internal hazard indices from emitted gamma rays were also calculated, and all values were found to be less than unity. The total absorbed dose rate (D) in nanogray per hour (nGyh⁻¹), resulting from exposure to gamma radiation (emitted by ^214^Pb and ^214^Bi, progeny of ^238^U, ^228^Ac, and ^208^Tl from ^232^Th, and contributions from ^40^K), was also calculated. In disperse dyes, the dose rate ranged from 14 to 49 nGyh⁻¹, in reactive dyes from 7.6 to 21 nGyh⁻¹, and in direct dyes from 7.2 to 8.8 nGyh⁻¹, all below the international recommended value of 55 nGyh⁻¹^25^. The annual equivalent dose rates for all samples were below the recommended limit of 1.5 mSvyear⁻¹ set by^26^. Additionally, the outdoor excess lifetime cancer risk (ELCRout) was calculated, with the highest average value observed in disperse dyes (0.19 ± 0.006), which is lower than the global average of 0.29^27^.

**Table 1:** Radiation hazard indices in the samples investigated.

Hazard indices	Definition	Formula
Radium equivalent Ra_eq_ (Bqkg^− 1^)	Radium equivalent activity (in Bqkg^− 1^) has been used to quantify radiation exposure to compare the specific activity of materials containing varying concentrations of ^226^Ra, ^232^Th, and ^40^K in a single value ^16,17^	Raeq = C_Ra_ + 1.43C_th_ + 0.077C_k_
Gamma index Iγ	Because of the excess external gamma radiation from surface material, the index Iγ has a correlation with the annual dosage rate ^18^	$I_{\gamma } = \frac{{C_{{Ra}} }}{{300}} + \frac{{C_{{Th}} }}{{200}} + \frac{{C_{K} }}{{3000}}$
External and internal hazard indices (H_ex_ and Hin)	Both the interior and exterior hazard indices (Hex and Hin) are used for predicting the effects of radiation on human health.	H_ex_ = C_Ra_/370 + C_Th_/259 + C_K_/4810 H_in_= C_Ra_/185 + C_Th_/259 + C_K_/4810
Absorbed dose rate Dair (nGy h 1)	Dose rate exposure to radiation sources in the air at one meter due to the concentrations (A) of ^238^U, ^232^Th, and ^40^K ^19,20^	D= [0.662 C_Th_ +0.427 C_U_ + 0.0432 C_K_]
Outdoor annual effective dose AEDin (mSv y 1)	Measure radiation exposure both indoors and outdoors during a one-year period ^21,22^	Effective dose rate (mSv/ year) = Dose rate (nGy h^− 1^) 8760 h x 0.2 × 0.7 Sv Gy^− 1^ x10^− 6^
Excess Lifetime Cancer Risk – (ELCR)	Chance of developing cancer in the long run for specific levels of exposure ^23,24^	ELCR _out_= E _out_ x 66 × 0.05

**Table 2:** Radiation hazards indices in different textile dyes.

Dye category	Samples	Ra_eq_	Iγ	H_ex_	H_in_	D(out)	E(out)	ELCR (out)
Disperse	Blue	48	0.35	0.13	0.15	18	0.02	0.07
	Red	54	0.38	0.14	0.19	20	0.02	0.08
	Yellow	65	0.47	0.17	0.24	27	0.03	0.011
	Black	50	0.36	0.13	0.14	17	0.02	0.07
	Orange	12	0.10	0.03	0.15	14	0.01	0.05
	Green	100	0.78	0.27	0.33	49	0.05	0.19
	Mean	55	0.40	0.14	0.2	24	0.02	0.08
Reactive	Yellow	50	0.36	0.13	0.12	15	0.02	0.06
	Blue	42	0.30	0.11	0.10	12	0.01	0.05
	Red	11	0.09	0.02	0.05	7.6	0.01	0.03
	Black	15	0.13	0.04	0.11	13	0.02	0.05
	Brown	7.9	0.06	0.02	0.15	13	0.01	0.05
	Orange	34	0.24	0.09	0.18	17	0.02	0.07
	Deep red	9.2	0.08	0.02	0.13	12	0.01	0.04
	Green	28	0.23	0.07	0.18	21	0.02	0.08
	Mean	25	0.19	0.06	0.13	13.82	0.01	0.05
Direct	Yellow	6.7	0.06	0.02	0.09	8.8	0.01	0.03
	Blue	17	0.13	0.05	0.07	7.2	0.01	0.03
	Red	31	0.22	0.08	0.05	8.0	0.01	0.03
	Dark red	20	0.14	0.05	0.08	7.9	0.01	0.03
	Mean	19	0.14	0.05	0.07	8.0	0.01	0.03

### Atomic absorption results

**Fig. 9:**
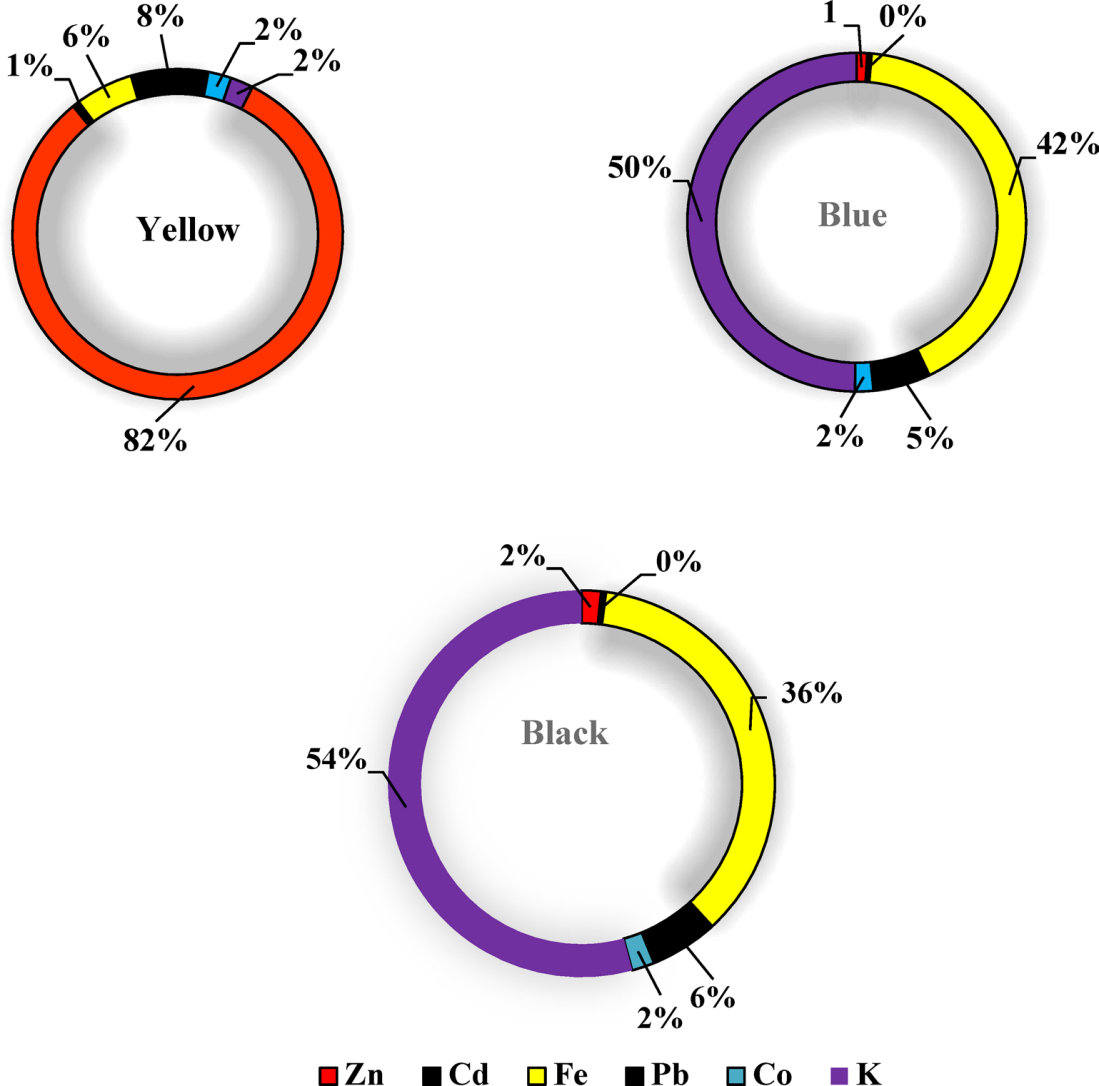
Toxic elements in reactive dyes.

**Fig. 10:**
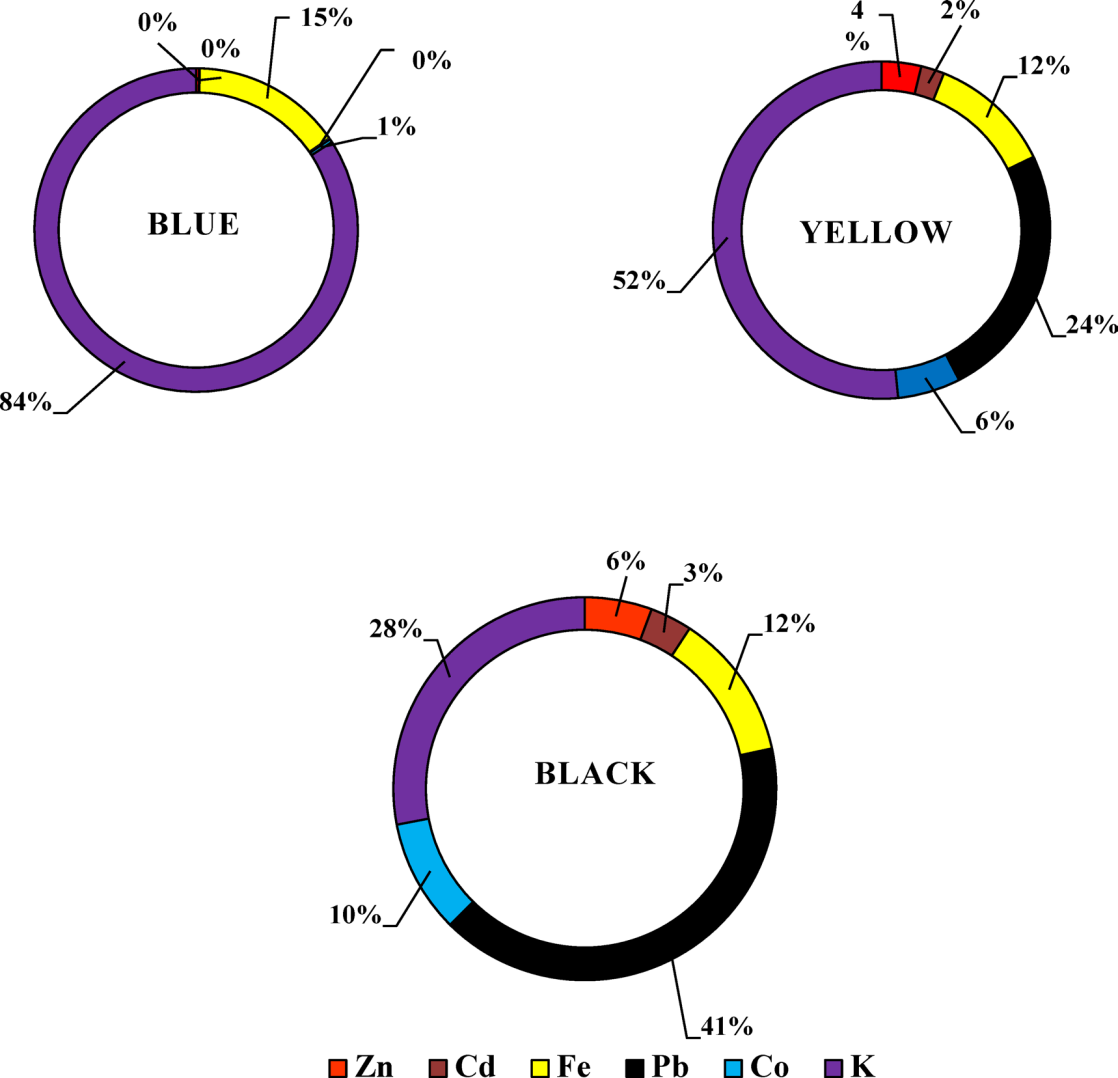
Toxic elements in disperse dyes.

**Fig. 11:**
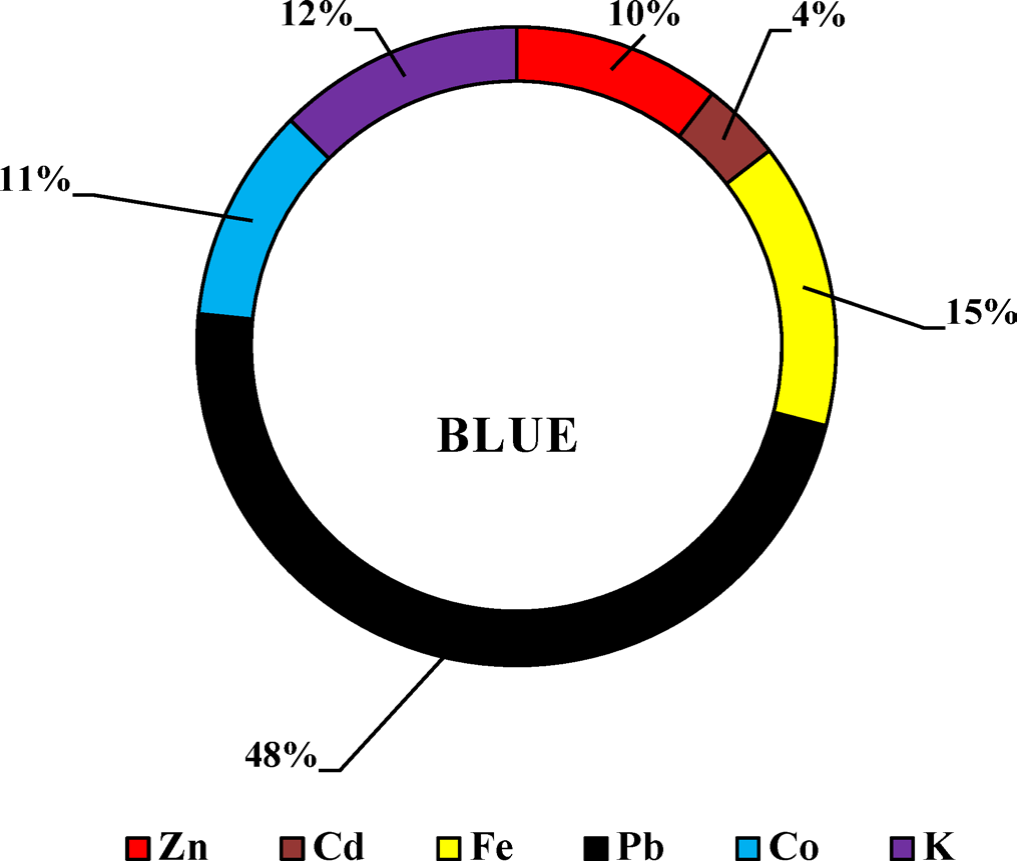
Toxic elements in direct dyes.

**Fig. 12:**
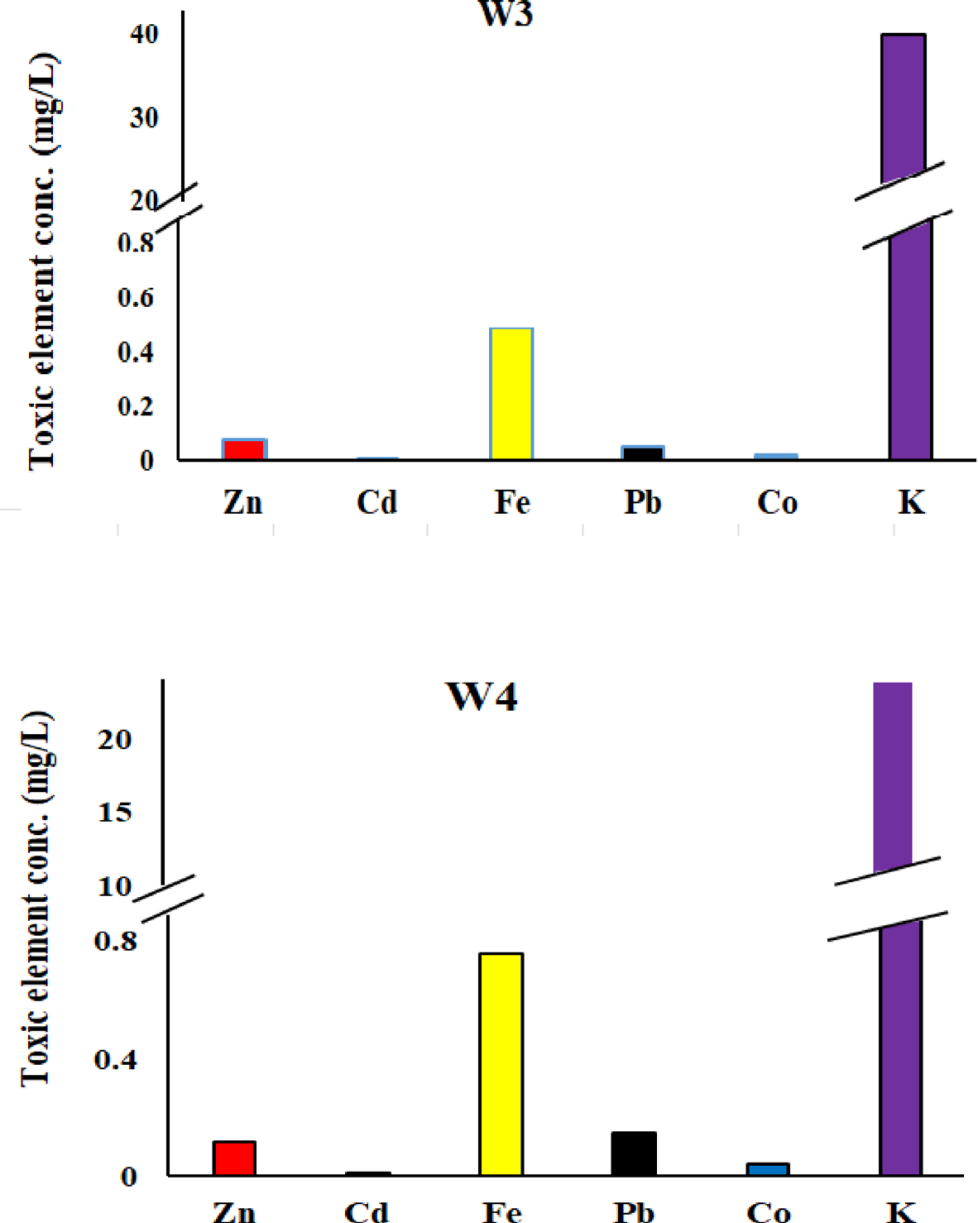
Toxic elements in Abour wastewater dyes.

**Fig. 13:**
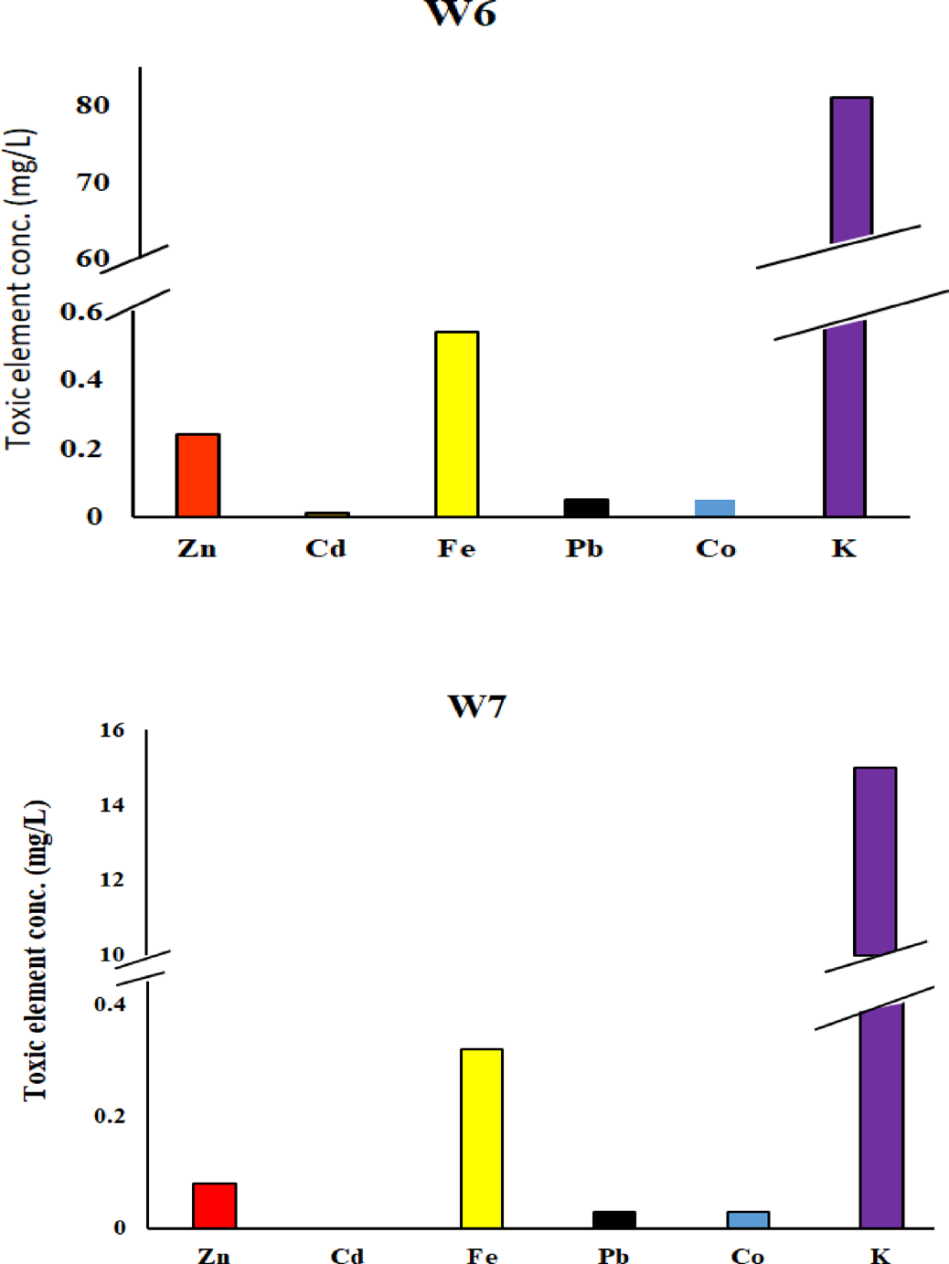
Toxic elements in Badr wastewater dyes.

Six heavy metals Zn, Cd, Fe, Pb, Co, and K—were identified in selected dye samples and dye wastewater (Tables 3 and 4). In reactive dyes, three color samples (yellow, blue, and black) were analyzed for these heavy metals. Zinc (Zn) and cadmium (Cd) showed their highest concentrations in yellow dyes, measuring 13 mg/kg and 0.11 mg/kg, respectively. Such levels are higher than the recommended levels 5 and 0.1 mg/kg according to OEKO-TEX^28^ respectively. Iron (Fe) and lead (Pb) had their highest values in blue dyes, with concentrations of 10 mg/kg and 1.32 mg/kg, respectively. These levels are higher than the recommended levels 1.00 and 0.2 mg/kg according to OEKO-TEX respectively. Potassium (K) ranged from 0.36 mg/L in yellow dye to 12 mg/kg in black dye Fig. 9. For disperse dyes, three samples—blue, yellow, and black—were also examined. Zn, Cd, Pb, and Co showed their highest concentrations in black dye, with values of 7.97 mg/kg, 4.98 mg/L, 57.28 mg/kg, and 13.45 mg/kg, respectively. These levels are higher than the permissible levels 5.00, 0.1, 0.2 and 1.0 mg/kg according to OEKO-TEX respectively. Fe and K reached their maximum levels in yellow dye, at 23.85 mg/kg and 104 mg/L, respectively Fig. 10. In the direct dye category, only blue dye was analyzed for these toxic elements. Zn, Cd, Fe, Pb, Co, and K were measured at 12.4 mg/kg, 4.77 mg/kg, 17.17 mg/kg, 56.29 mg/kg, 12.88 mg/kg, and 14.79 mg/kg, respectively Fig. 11. These heavy metals can leach into water bodies during dyeing processes and through the disposal of textile waste, leading to water pollution and negatively impacting aquatic ecosystems. In this study, dye wastewater was analyzed for heavy metals in two cities, Obour and Badr. The results indicated that the average levels of zinc, cobalt, and potassium were higher in Bader city compared to Obour city, whereas cadmium, iron, and lead concentrations were higher in Obour city Figs. 12 and 13.

**Table 3:** Toxic elements (mgL^− 1^) in some textile dye samples.

Dye category	Samples	Zn	Cd	Fe	Pb	Co	K
Reactive	Yellow	13	0.11	0.9	1.2	0.36	0.36
	Blue	0.24	0.108	10	1.32	0.42	12
	Black	0.36	0.09	8	1.26	0.42	12
Disperse	Blue	0.3	0.06	15	0.12	0.33	84
	Yellow	7.65	4.5	23.85	49.51	12.15	104
	Black	7.97	4.98	17.43	57.28	13.45	39
Direct	Blue	12.4	4.77	17.17	56.29	12.88	14.79

**Table 4:** Toxic elements (mgL^− 1^) in some dyes wastewater samples.

Wastewater Source	Samples	Zn	Cd	Fe	Pb	Co	K
Obour	W3	0.08	0.007	0.49	0.05	0.02	40
	W4	0.12	0.01	0.76	0.15	0.04	24
Bader	W6	0.24	0.007	0.54	0.05	0.05	81
	W7	0.08	0.003	0.32	0.03	0.03	15

## Discussion

The pH, chemical interactions, and geological formations of the raw materials from which the samples were derived could be among the reasons for the differences in activity levels between the samples^29^^30^. In previous research, we measured the activity concentrations of ^238^U, ^232^Th, and ^40^K in nine textile industry dyes using gamma spectrometry with a Hyper Pure Germanium (HPGe) detector. The mean activity concentrations were 29.37 ± 4.48 Bq/kg for ^238^U, 1.15 ± 0.13 Bq/kg for ^232^Th, and 565 ± 4 Bq/kg for ^40^K (Abdel Ghany & Ibrahim, 2014)^9^. Additionally, we measured radon and thoron concentrations (6978 ± 2491 Bq/m³ and 3457 ± 996 Bq/m³, respectively). The mean activity concentrations in previous studies were higher than those found in the present work (This discrepancy may be attributed to differences in raw materials, manufacturing processes, and the specific types of textile dyes used, which can influence radionuclide content. Other contributing factors may include geological variations in the source materials, differences in sample preparation techniques, and variations in measurement conditions), where the values were 19.14 ± 5.1, 8.08 ± 2.2, and 276 ± 37 Bq/kg in disperse dyes; 8.82 ± 2.0, 3.23 ± 0.1, and 80.06 ± 10 Bq/kg in direct dyes; and 13.97 ± 3.7, 3.96 ± 0.1, and 153.36 ± 20 Bq/kg in reactive dyes. This Data confirm that the activity concentrations of ^238^U, ^232^Th, and ^40^K for all samples under investigation are lower than recommended values 33, 32, and 412 Bq/kg respectively^31^. These findings suggest that textile dyes may contain measurable levels of radioactivity, which should be taken into consideration. In this study, we analyzed different categories of textile dyes, including reactive, disperse, and direct dyes. The radiation hazard indices for all categories were within the recommended safety levels. Long-term exposure to radiological contaminants in textile dyes, especially those containing naturally occurring radioactive materials (NORM), can lead to several health risks. These risks arise from both external radiation exposure (from direct contact with contaminated materials) and internal exposure (through inhalation or ingestion of radioactive particles). Some potential long-term health effects include increased cancer risk, organ damage, genetic and reproductive effects, respiratory and skin disorders, and neurological Effects. We also calculated the radiation hazard indices in dye wastewater from Obour and Badr cities. The results showed that (i) the radiation hazard indices in Obour city were higher than those in Badr city) Several geographical and geochemical factors may explain radiation hazard differences between Obour and Badr cities. Obour could be built on bedrock or soil with higher radioactive element concentrations, while Badr may have lower-radiation sedimentary formations. Granitic or phosphate-rich soils in Obour might elevate radiation levels, whereas Badr’s geology may contain fewer radionuclides. Soil porosity and permeability influence radon exhalation—Obour’s fractured rocks or sandy soils may enhance radon release, while Badr’s denser soils could trap it. Industrial activities in Obour, such as phosphate fertilizer use or mining, may contribute to radiation, whereas Badr might have fewer such sources. Additionally, altitude and climate differences could impact radon accumulation, with lower-lying areas in Obour trapping more radon, while Badr’s higher elevation or windier conditions may aid dispersion (, and (ii) all recorded values were below permissible levels (Table 5**)**. Heavy metals such as zinc, cadmium, iron, lead, cobalt, and potassium are often present in textile dyes and are used to achieve specific colors or properties in fabrics^32^. When found in high concentrations, these metals may pose health risks through skin absorption potentially leading to changes in skin microbiota, dermatitis, irritation, and allergies^33^. Compared to other studies, the mean concentrations of Pb, Zn, Fe, and Cd in the present study are 23.85, 5.98, 13.19, and 2.08 mg/kg, respectively. In contrast, a study conducted in India reported significantly lower concentrations of 0.056, 0.34, 0.61, and 0.01 mg/kg, respectively^34^. These findings indicate that the heavy metal levels in the current study are considerably higher. Therefore, strict precautions must be implemented to protect both workers and the public. Another major concern in the textile dye industry is wastewater, which can become an environmental hazard due to the presence of heavy metal ions like Zn, Cd, Fe, Pb, Co, and K. These ions can have severe toxicological effects on the environment and pose significant health risks to humans such as bioaccumulation occurs when heavy metals persist in biological systems, accumulating in tissues over time due to slow elimination. Skin exposure to textile dyes containing heavy metals can lead to gradual absorption and deposition in organs. Key concerns include Cadmium (Cd): Bioaccumulates in kidneys and liver, disrupting enzyme activity and leading to renal toxicity. Lead (Pb): Deposits in bones, brain, and soft tissues, affecting neurological and cognitive functions. Zinc (Zn) & Iron (Fe): Essential trace elements but toxic in excess, accumulating in the liver and causing oxidative stress. Liver and causing oxidative stress. While heavy metals enhance textile dye properties, their potential bioaccumulation and toxic effects necessitate strict monitoring and safer alternatives^35^. Future research should focus on developing eco-friendly, non-toxic dye alternatives while ensuring compliance with safety standards.

**Table 5:** Radiation hazards indices in dyes wastewater.

Wastewater source	Samples	Ra_eq_	Iγ	H_ex_	H_in_	D(out)	E(out)	ELCR (out)
Obour	W1	36	0.28	0.09	0.14	16.83	0.02	0.07
	W2	11	0.09	0.03	0.08	9.80	0.01	0.04
	W3	12	0.09	0.03	0.10	10.9	0.01	0.04
	W4	13	0.10	0.03	0.09	10.9	0.01	0.04
	Mean	18	0.07	0.04	0.10	12.0	0.01	0.05
Badr	W5	11	0.09	0.03	0.09	10.03	0.01	0.04
	W6	12	0.09	0.03	0.10	10.68	0.01	0.04
	W7	12	0.01	0.03	0.08	10.05	0.01	0.04
	W8	13	0.10	0.04	0.10	11.38	0.01	0.04
	Mean	12	0.07	0.03	0.09	10.53	0.01	0.04

## Conclusions

This study assesses the natural radioactivity levels (^238^U, ^226^Ra, ^232^Th, and ^40^K) and associates radiological hazards in three types of textile dyes: disperse, direct, and reactive. The findings indicate that the specific radioactivity is highest in disperse dyes (due to the raw materials, rare earth elements, mineral-based pigments, and industrial processing conditions). Additionally, the presence of heavy metals in textile dyes poses serious environmental and health risks. These elements can contaminate water bodies during dyeing processes and through the disposal of textile waste, leading to water pollution and harmful effects on aquatic life. Improper disposal of dye-laden waste can also result in soil contamination, impacting plant life and potentially entering the food chain. To mitigate these risks, stricter industry regulations should be implemented to monitor and limit the concentration of radioactive elements and heavy metals in textile dyes. Regulatory agencies should establish permissible limits for radionuclide content in dyes, enforce waste management protocols, and promote eco-friendly dyeing technologies. Additionally, industries should adopt advanced wastewater treatment techniques, such as adsorption and membrane filtration, to minimize environmental contamination. Future research should focus on developing sustainable and low-radioactivity dye alternatives, assessing long-term environmental impacts, and exploring efficient remediation methods for contaminated sites. Further investigations are also needed to evaluate the potential bioaccumulation of radioactive elements and heavy metals in the food chain, ensuring comprehensive risk assessments and public health protection.

## Data Availability

Data sharing is not applicable to this article where the manuscript includes all the data we generated.
